# Supplementary figures and images for: Interspecies microbiome transplantation recapitulates microbial acquisition in mosquitoes
Source: Microbiome. 2022 Apr 11;10:58. doi: 10.1186/s40168-022-01256-5 (PMC8996512; doi:10.1186/s40168-022-01256-5)

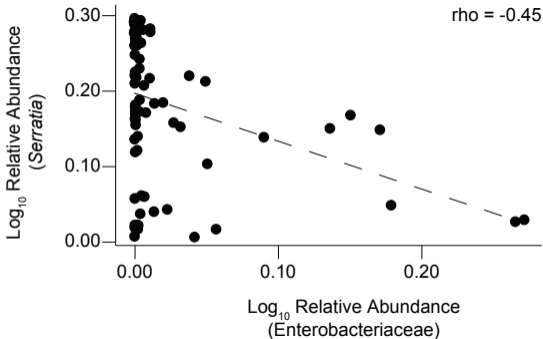

Supplement: Supplementary file 4 — Additional file 3: Supplementary Figure 1. Significantly negative correlation between relative abundance of ASVs belonging to the bacterial family Enterobacteriaceae and ASVs belonging to the genus Serratia across all Ae. aegypti and Cx. quinquefasciatus donor individuals (Spearman’s rank test, P < 0.05). [file 40168_2022_1256_MOESM3_ESM.pdf]

Donor → Recipient = *Ae. aegypti* → *Ae. aegypti*

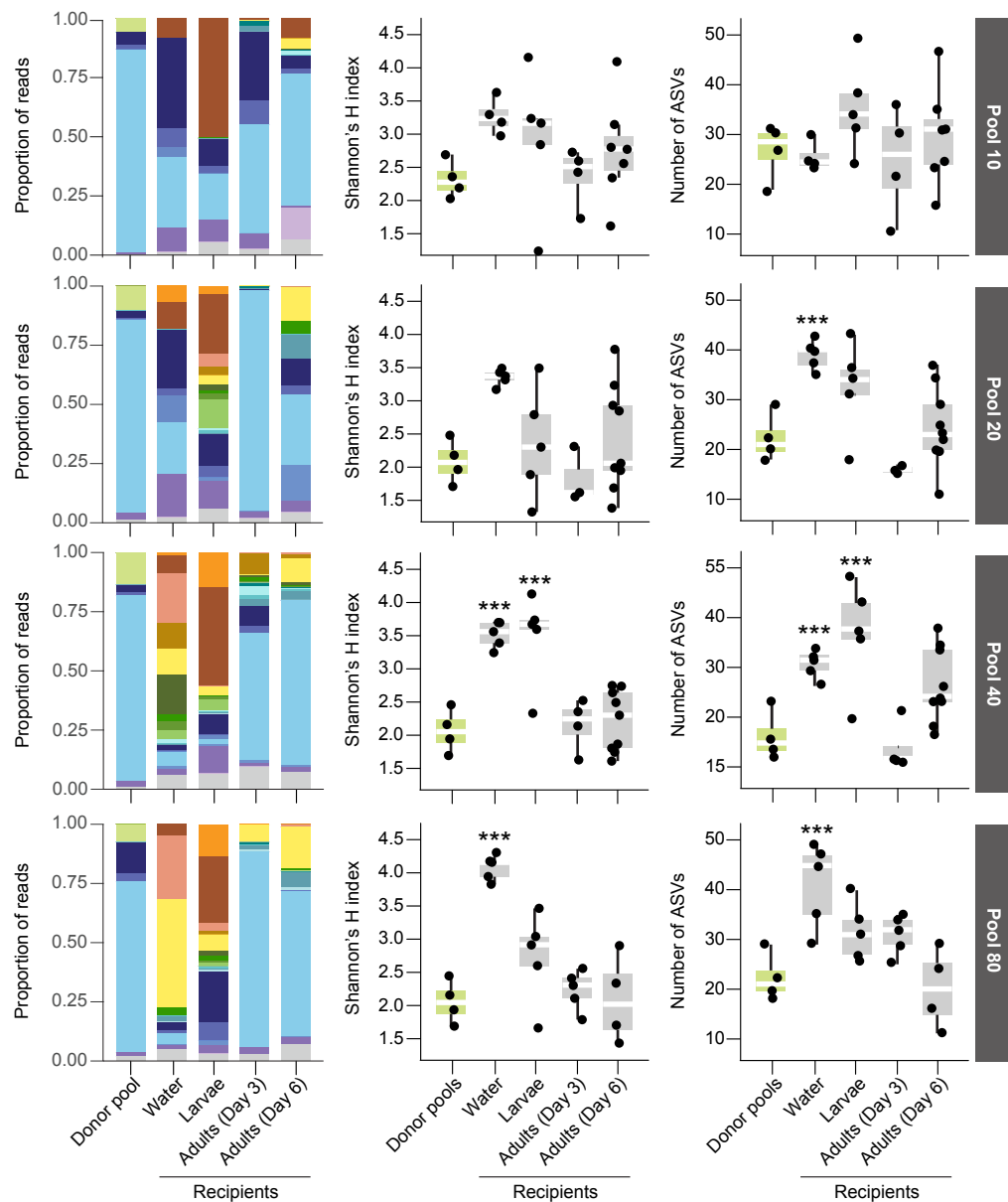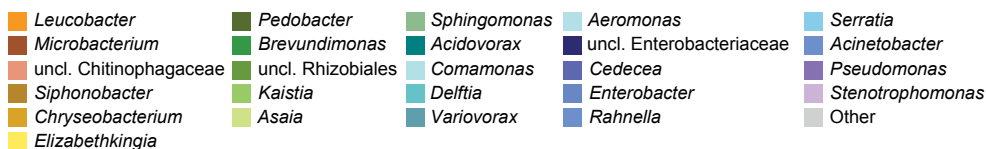

Supplement: Supplementary file 5 — Additional file 4: Supplementary Figure 2. (Left) Relative abundance of bacterial genera in Ae. aegypti donor pools and recipient samples. Biological replicates were pooled for the bar graphs presented. Low abundance genera (<1%) are represented by the ‘Other’ category. (Right) Alpha diversity of donor pools and recipient samples, as measured by Shannon’s H index and ASV richness. Box-and-whisker plots show high, low, and median values, with lower and upper edges of each box denoting first and third quartiles, respectively. Asterisks (***) indicate samples that significantly differed from the donor pools (Dunn’s test with Bonferroni correction, P < 0.0125). [file 40168_2022_1256_MOESM4_ESM.pdf]

Donor → Recipient = *Cx. quinquefasciatus* → *Ae. aegypti*

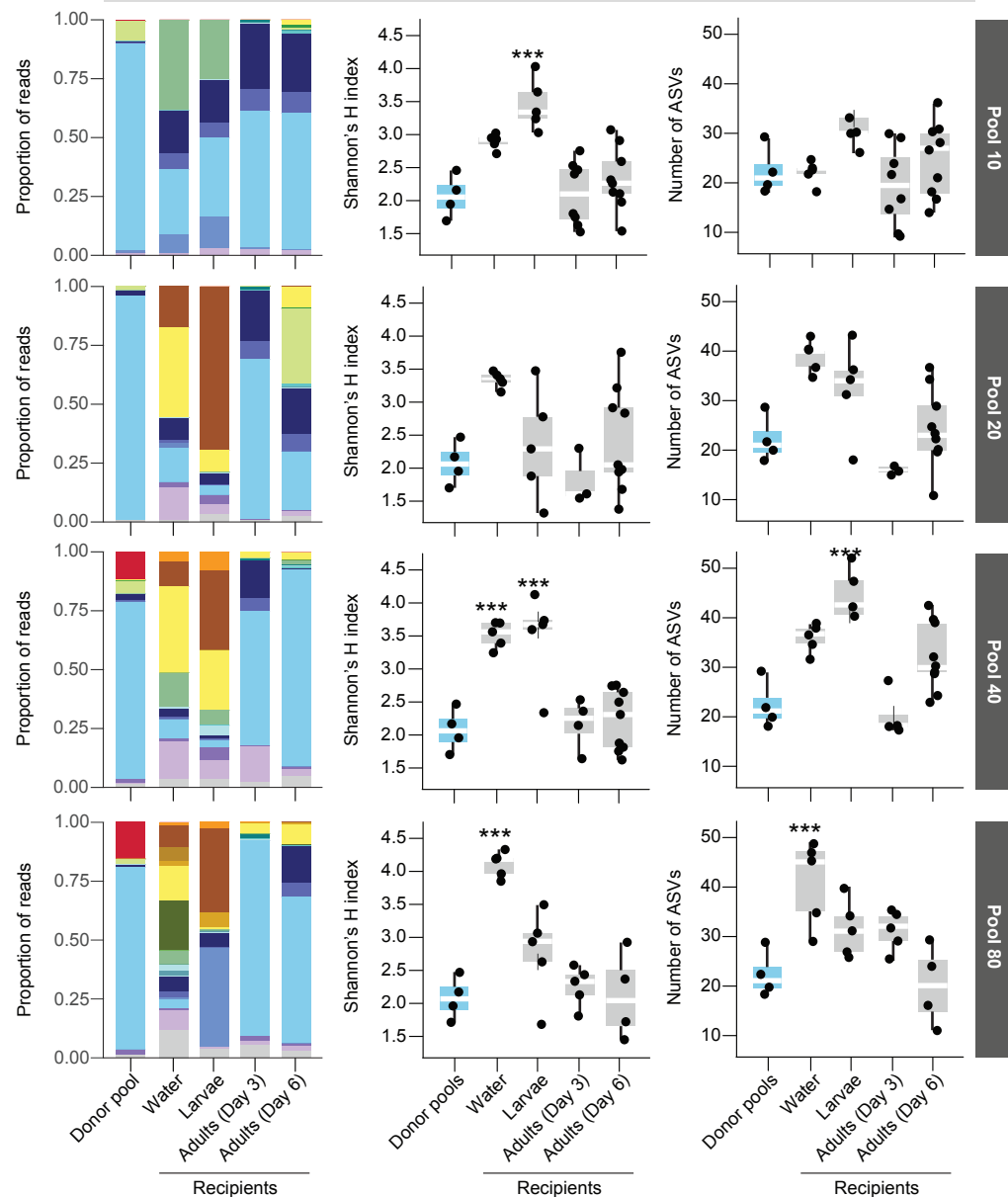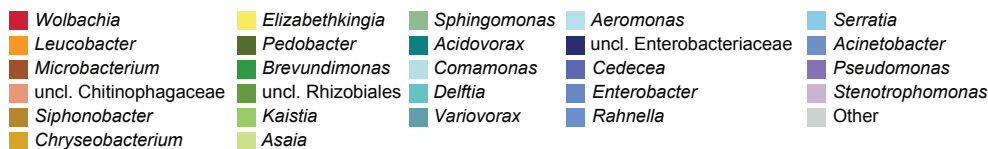

Supplement: Supplementary file 6 — Additional file 5: Supplementary Figure 3. (Left) Relative abundance of bacterial genera in Cx. quinquefasciatus donor pools and recipient samples. Biological replicates were pooled for the bar graphs presented. Low abundance genera (<1%) are represented by the ‘Other’ category. (Right) Alpha diversity of donor pools and recipient samples, as measured by Shannon’s H index and ASV richness. Box-and-whisker plots show high, low, and median values, with lower and upper edges of each box denoting first and third quartiles, respectively. Asterisks (***) indicate samples that significantly differed from the donor pools (Dunn’s test with Bonferroni correction, P < 0.0125). [file 40168_2022_1256_MOESM5_ESM.pdf]

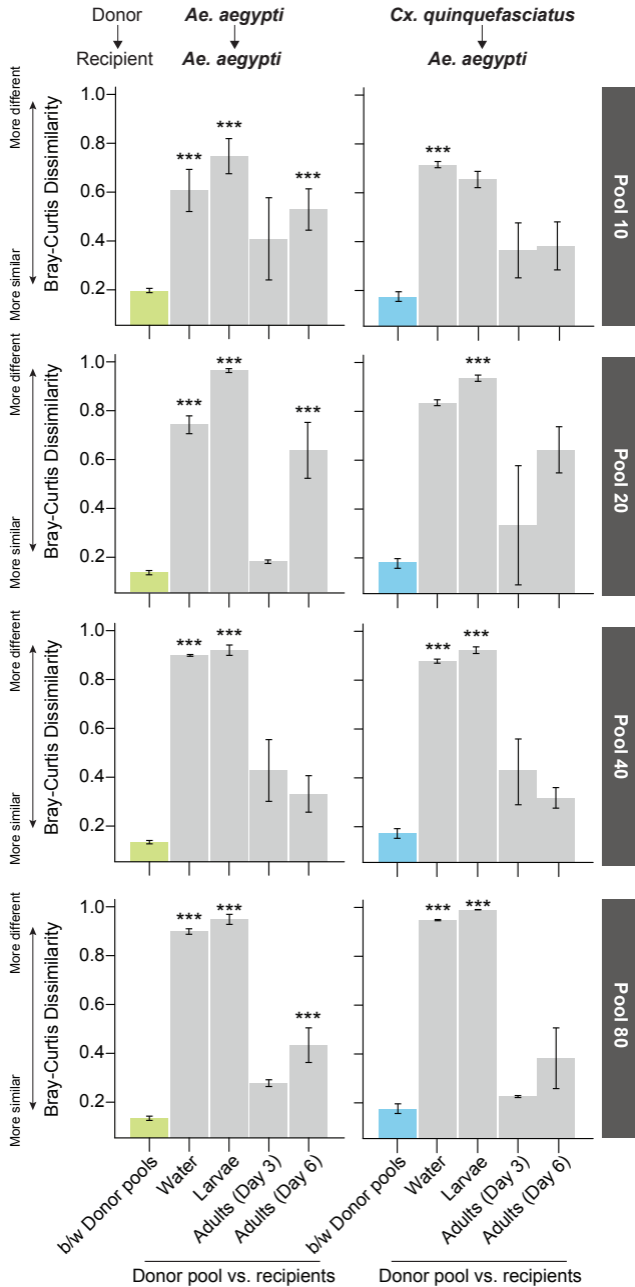

Supplement: Supplementary file 7 — Additional file 6: Supplementary Figure 4. Average Bray-Curtis dissimilarity between (b/w) donor pools versus between a given donor pool and recipient samples. Mean values ± standard errors are shown. Asterisks (***) indicate comparisons for which the average dissimilarity between a given donor pool and group of recipient samples was significantly higher than that expected as a result of the transplantation procedure itself (i.e., between donor pools) (Dunn’s test with Bonferroni correction, P < 0.0125). [file 40168_2022_1256_MOESM6_ESM.pdf]

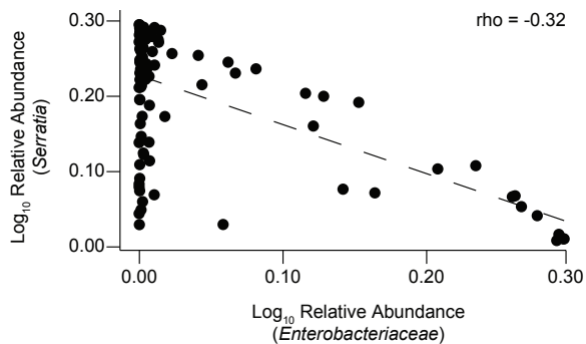

Supplement: Supplementary file 8 — Additional file 7: Supplementary Figure 5. Significantly negative correlation between relative abundance of ASVs belonging to the bacterial family Enterobacteriaceae and ASVs belonging to the genus Serratia across all recipient Ae. aegypti adults (Spearman’s rank test, P < 0.05). [file 40168_2022_1256_MOESM7_ESM.pdf]
